# Effect of THI on Milk Production, Percentage of Milking Cows, and Time Lying in Holstein Cows in Northern-Arid Mexico

**DOI:** 10.3390/ani13101715

**Published:** 2023-05-22

**Authors:** Rafael Rodriguez-Venegas, Cesar Alberto Meza-Herrera, Pedro Antonio Robles-Trillo, Oscar Angel-Garcia, Martín Alfredo Legarreta-Gonzalez, Humberto Filemón Sánchez-Vocanegra, Rafael Rodriguez-Martinez

**Affiliations:** 1 Programa de Doctorado en Ciencias Agropecuarias Unidad Laguna, Universidad Autónoma Agraria Antonio Narro, Torreón 27054, Coahuila, Mexico; rafar.v.v@gmail.com; 2 Unidad Regional Universitaria de Zonas Áridas, Universidad Autónoma Chapingo, Bermejillo 35230, Durango, Mexico; cmeza2020@hotmail.com; 3 Unidad Laguna, Departamento de Producción Animal, Universidad Autónoma Agraria Antonio Narro, Torreón 27054, Coahuila, Mexico; 4 Unidad Laguna, Departamento de Ciencias Médico Veterinarias, Universidad Autónoma Agraria Antonio Narro, Torreón 27054, Coahuila, Mexico; mvz.oscar_2207@hotmail.com; 5 Asignatura e Investigador, Universidad Tecnologica de la Tarahumara, Guachochi 33180, Chihuahua, Mexico; mlegarreta@uttarahumara.edu.mx; 6 Private Consultant, Torreón 27015, Coahuila, Mexico; hsanchezv63@gmail.com

**Keywords:** heat stress, dairy cows, arid North Mexico, milk production, milk composition, economic losses

## Abstract

**Simple Summary:**

The main dairy area of the country is in the arid central north of Mexico (25° NL). It is characterized by a dry climate with high temperatures, where dairy cattle are subject to prolonged periods of heat stress (HS). Due to this, through the THI and the year’s seasons, the effect of HS upon milk production, feed-to-milk efficiency, and cow comfort was evaluated in 2467 Holstein–Friesian cows between 2016 and 2019 in an intensive dairy management system. Total milk production decreased as THI increased, while milk composition also suffered changes due to HS. The percentage of cows in production and cows’ lying time exhibited a visible drop from a THI of 68–71. Differences were also observed across seasons, with the highest milk production values in Winter and Spring and the lowest in Summer. In the same way, lying time differed among seasons, with a longer resting time in Winter and less time in Summer. Finally, the potential economic burden that HS caused at the producer and industry-market levels and its impact on nutrient and alimentary security at the societal level were also quantified.

**Abstract:**

The possible effect of heat stress (HS), measured with the temperature–humidity index (THI) across seasons of the year (SY) upon milk production (MP), feed-to-milk efficiency (FME), and cow comfort (CC) was assessed in Holstein–Friesian cows in northern-arid Mexico. Data from 2467 cows (2146 milking and 321 dry) were recorded across SY [spring (SP), summer (SM), autumn (AT), and winter (WN)] between 2016 and 2019 in an intensive dairy farm located in the Comarca Lagunera (25° NL) with large fluctuations regarding ambient temperature and solar radiation. The THI was stratified into four classes: non-HS, <68; light HS, 68–71; moderate HS, 72–76; and intense HS, ≥77. The considered response variables were Milk production: both on a farm basis (totMP) and on a cow basis (cowMP); Nutritional efficiency: dry matter intake (DMI, kg); Feed conversion efficiency (FCE, kg) and energy-corrected milk (ECM, kg); Percentage of milking cows: (MC%); and Cow comfort: lying time (LT, h). Analyses of variance for unbalanced data were performed through “R”. Both totMP and cowMP differed (*p* < 0.05) as HS increased; the largest values (i.e., 77,886 L and 35.9 L) occurred at lower THIs (i.e., <68 and 68–71) while the milk production fell (i.e., 66,584 L and 31.7 L) with the highest THIs (i.e., ≥77). Not only feed-to-milk efficiency (i.e., DMI, FCE, and ECM) but also the MC% exhibited a similar trend; a visible drop (*p* < 0.05) occurred from a THI of 68–71 onwards. Furthermore, the LT declined as the THI augmented, from 10.6 h at <68 to 8.5 h at ≥77. Moreover, differences (*p* < 0.05) also arose across seasons; TotMP, cowMP, DMI, FCE, and ECM revealed their largest (*p* < 0.05) values in WN and SP, halfway ones in AT, with the lowermost figures in SM. In the same way, cow comfort differed (*p* < 0.05) among seasons, with diverse lying times (h); WT, 10.5; AT, 10.20; SP, 9.3 h; and 8.8 in SM. Finally, the potential economic burden that HS caused at the producer (USD 233.2 million) and industry-market levels (USD 311.1 M), as well as its impact upon nutrient and alimentary security at the society level (i.e., 311 M milk liters and 195,415.82 Gcal), were also quantified.

## 1. Introduction

One of the main external factors that can negatively affect the performance of dairy cows is the thermal environment in which they live [1]. In fact, high performance animals with high genetic merit are particularly sensitive to heat stress (HS) because of their augmented thermogenesis due to their higher metabolic activity [2,3]. In dairy cows increasing their milk production from 35 to 45 kg/d, the HS temperature threshold may be decreased by 5 °C, meaning that cows will become heat stressed earlier [4]. HS conditions represent a significant financial burden, estimated in the US to be between USD 897 and 1500 million annually; such economic losses occur because most of the animals are raised in places where environmental temperatures are outside the thermoneutral comfort zone [3].

Heat stress affects the ability of animals to thermoregulate and augments thermogenesis while decreasing feed intake, fertility, and milk production [5,6,7,8,9,10,11], causing significant and adverse implications for livestock productivity [12]. This can greatly decrease in such a way that it is considered that HS represents a significant financial burden, estimated in the US as between USD 897 and 1500 million annually [3]. However, not only is milk yield loss observed, but there are associated changes in milk components, including protein [13], fat, solid non-fat, casein and lactose contents, milk urea, and somatic cell score [14]. Besides productive and reproductive impairments, animal well-being has also been described, such as lying time, a very important behavioral characteristic that is a key marker of dairy cows’ physiological and health status [15,16]. Therefore, the cow’s lying time can be used as an indicator of the cow´s well-being [17]. Another interesting indicator, although not commonly used when evaluating the effect of HS on farm productivity, is the percentage of cows milking with respect to dry cows across a defined period, usually on an annual basis [18].

The Temperature Humidity Index (THI) is a useful tool to measure the productive response as a function of climate [19,20,21,22]. It is based on air temperature and relative humidity, serving as a measure of the sum of external forces on the animal that acts to displace body temperature from its homeostatic point [22]. Although it was common to place the THI threshold at 72 as the point where milk synthesis begins to decline, and other indicators such a milk composition and feed intake are altered [19,23], other data indicate that high-yielding dairy cows reduce their milk production with a THI of around 68 [24,25], or for other conditions [26].

We recently carried out a study [27] on HS conditions (THI) and seasonal performance in the region that produces the largest amount of cow’s milk in Mexico, the Comarca Lagunera, using climatic Big Data obtained from five farms inside the regional area and for a period of 5 years, observing that HS conditions occur in the region during most of the year (305 d). The results generated the need to investigate how HS affects the dairy industry and consumers. Therefore, this study aimed to quantify the possible effect of different THI levels on milk production and milk composition, feed-to-milk efficiency, and animal comfort of dairy cows in northern-arid Mexico. Moreover, we aimed to quantify the potential economic burden that HS would cause at farm, regional, and societal levels.

## 2. Materials and Methods

The Comarca Lagunera (CL), a very important agro-industrial region located in central northern-arid Mexico, generates 21% of the Mexican national milk volume. Nonetheless, it is characterized by low precipitation (i.e., 200 mm yearly) and significant fluctuations in monthly average temperatures (i.e., 12.7 °C in January, 28.5 °C in June), with extremes from −5 °C to 41.5 °C, with extensive solar radiation.

### 2.1. Study Design

This was an observational, retrospective, longitudinal, and comparative study. The observational unit were farm daily liters of milk. The data, both climatic and productive, were obtained from an intensive dairy farm (25° 89′ NL and 103° 22′ WL).

### 2.2. Dairy Farm, Population Cows, and Climatic Data

The period of data analyzed was 2016–2019, and the average cow population during the period of study was 2467 Holstein cows (2146 milking and 321 dry ones), housed in pens for 120 animals and with 11 m^2^ of shaded area per cow, and fed twice a day with a total mixed ration (TMR). The observational unit was the daily milk production of the farm. Years were divided into seasons: spring (21 March–20 June), summer (21 June–20 September), autumn (21 September–20 December), and winter (21 December–20 March).

Ambient temperature (°C) and relative humidity (%) data were obtained from the meteorological station situated in an intensive dairy farm (25° 89′ NL and 103° 22′ WL) in the CL. The THI was calculated as (1.8·T + 32) − [(0.55 − (0.0055 × RH) ((1.8 × T) − 26)] [27] and classified into four levels: without HS (WoHS = <68 THI); light HS (LHS = 68–71 THI); moderate HS (MHS = 72–76 THI), and intense HS (IHS = ≥77). Categorized THI and seasons were the independent variables.

The effect of the levels THI and season on the performance and comfort variables were compared on the next dependable variables:

Milk performance: The volume of daily milk produced at the farm (totMP) was calculated using Afi Farm 5.5™, Afimilk, Afikim, Israel, and average daily milk performance was calculated at cow level (cowMP).

Milk composition: percentages of fat and protein in milk and amount of urea in milk were obtained by infrared spectroscopy (LactoScope™ 300 FT-IR Analyzer Perkin Elmer, Waltham, MA, USA).

Feed-to-milk efficiency: The diet’s average dry matter intake (DMI) was determined through daily weighing of the feed offered and orts in the following days. The amount of feed offered was adjusted weekly so that there were approximately ten per cent orts [28]; Feed conversion efficiency (FCE) was calculated as kg of milk/kg of DMI [29]; and Energy-Corrected Milk (ECM) was calculated as (12.82 × kg fat) + (7.13 × kg protein) + (0.323 × kg milk) [30].

Economic values: Based on the annual average of the economic input received by the producer (i.e., EIP), we also projected the financial burden (i.e., EB) generated by the HS not only at the farm-producer level (i.e., EBT) but also at the regional level (i.e., CL; EBCL) expressed as USD. We also projected the loss that said thermal insult generates with respect to the reduction in the milk supply to society, expressed not only as milk volume but also as kg of fat and protein. Moreover, to quantify the concomitant impact of HS upon milk performance, the chemical values proposed by Nayak et al. [31] were considered to escalate such HS insult from an animal viewpoint up to farm and regional levels. The following milk chemical information involved in the performed analyses considered water (87.8%), fat (3.6%), protein (3.2%), lactose (4.7%), and energy (64.0 kcal) as suggested by Nayak et al. [31].

Percentage of milking cows: (MC% = cow milking × 100/total of cows).

Cow comfort: Defined as the number of daily hours at rest; lying time (LT, h; measured through a pedometer Afi Act II™, Enosburg Falls, VT, USA).

### 2.3. Statistical Analyses

The effect of HS level and season of the year on milk performance, milk composition, digestive efficiency, and comfort variables were evaluated by R (R Core Teams, Vienna, Austria, 2022) using the base program for the Analysis of Variance (ANOVA), considering a *p* < 0.05 as a statistical difference for all the results obtained, and the library emmeans [31] to obtain estimated marginal means (EMMs) as suggested, by Searle, Speed, and Milliken [32] for pairwise comparisons. The results are presented as lsmeans ± standard error, and the statistical difference was considered at *p* < 0.05.

## 3. Results

### 3.1. Descriptive Statistics

Table 1 shows the descriptive statistics for the analyzed variables in the study.

### 3.2. Effect of HS Level

#### 3.2.1. Milk Performance, at either the Farm Level or the Cow Level

TotMP was affected by THI. No differences were found (*p* > 0.05) in TotMP between <68 and 68–71 levels, with a daily average of 77,500 L. However, a decline in milk production occurred as the THI increased (*p* < 0.05) in the different THI classes, 72–76 and ≥77 (78,886 ± 985.5 L, 75,095 ± 586 L, and 66,584 ± 870 L), respectively (Table 1). A similar trend occurred with CowMP; the highest (*p* < 0.05) milk production occurred at <68 and 68–71 THI levels with an average of 35.9 L, while the CowMP decreased (*p* < 0.05) at THIs 72–76, and ≥77 levels, with milk production of 34.37 ± 0.17 L, and 31.79 ± 0.26 L, respectively (Table 2).

#### 3.2.2. Feed-to-Milk Efficiency

According to the DMI, no differences (*p* < 0.05) were observed in cows at THIs < 68 and 68–71 (i.e., avg = 23.7 kg). The lowest DMI (*p* < 0.02) occurred at 68–71 THI (22.76 ± 0.11 kg) and at ≥77 THI (21.59 ± 0.16 kg) (Table 1). Concerning the FCE (Table 1), no differences (*p* > 0.05) arose among the THIs < 68, 68–71, and 72–76 (i.e., DMI-avg. 1.57 units], while the ≥77 groups had the lowest (*p* < 0.05) FCE, with 1.51 ± 0.01 units). The ECM was higher (*p* < 0.05) at both <68 and 68–71 THIs (i.e., avg. 35.66 kg), registering a noticeable drop as the THI increased from 68–71 onwards (Table 1).

#### 3.2.3. Percentage of Milking Cows (%MC)

In the case of milking cows (Table 1), a negative (*p* < 0.05) relationship between THI and milking cows occurred. As the THI increased, the proportion of milking cows declined (THI 68–71 and 89.1 ± 0.61%; THI 72–71, 87.11 ± 0.36 %, and THI ≥ 77, 84.0 ± 0.54%).

#### 3.2.4. Cow Comfort (Daily Average Lying Time per Cow)

Regarding the LT (Table 1), the greater the THI, the less time a cow lies down. The lying time observed values along with the THIs were: <68, and 68–71 THI, the average lying time was 10.62 h; with THI 68–71, we observed 10.53 h, while with THI 72–76 it was 9.46 h and, for THI ≥ 77, the lying time was reduced to 8.52 ± 0.13 h.

#### 3.2.5. Milk Composition (Milk Fat, Protein Fat, Fat/Protein Milk Ratio, Milk Urea)

Milk fat was affected by HS level, showing the lowest percentage of fat content (3.33 ± 0.02 at <68 THI), while the other HS levels had an average of 3.42% of fat. Concerning protein milk, while the THI levels were increasing, the percentage of milk protein descended (from 3.19 ± 0.01 at <68 THI to 3.09 ± 0.003 at ≥77 THI). The fat/protein milk ratio was higher at ≥77 THI, and the lowest value was found at 68–71 and 72–76 THI (1.08 ± 0.003). The highest milk urea value (11.0 ± 0.13) was also found at ≥77 THI (11.0 ± 0.13), with the lowest one at 68–71 THI.

### 3.3. Effect of THI and Season

#### 3.3.1. Milk Production at the Farm Level and per Cow

The TotMP and cowMP showed differences (*p* < 0.05) across seasons; differences were quantified in both variables where the highest values occurred in winter and spring, while an intermediate production occurred in autumn, with the lowest production observed in summer; the last was true for both TotMP and cowMP (Table 3).

#### 3.3.2. Dry Matter Intake, Feed Conversion Efficiency, and Energy-Corrected Milk

The DMI denoted differences (*p* < 0.05) across seasons (Table 2); the highest value occurred in winter (24.23 kg), intermediated values were observed both in spring (23.29 kg) and autumn (22.79 kg), while the lowest value was detected in summer (21.18 kg), all of them with a SE of 0.06. As for the FCE, differences across seasons were also detected, with the highest values (*p* < 0.05) observed in spring and winter (1.56 units, SE 0.01), while the lowest ones (*p* < 0.05) occurred in summer and autumn (1.53 units, SE 0.01; Table 2). Moreover, the ECM (Table 2) denoted different lsmeans across seasons, showing corresponding values for winter, spring, autumn, and summer of 36.4, 34.9, 34.0, and 31.2 ± 0.20 kg respectively.

#### 3.3.3. Percentage of Milking Cows

Apropos, the response variable milking cows denoted that the better (*p* < 0.05) values occurred during spring and winter (avg. 91.05 ± 0.20%); an intermediate value was registered in autumn, while the lowest value was recorded in summer; 84.63% and 80.82%, ± 0.23, respectively (Table 2).

#### 3.3.4. Cow Comfort and Wellness Expressed as the Average Lying Time per Cow

The comfort values unveiled differences (*p* < 0.05) across seasons. The values obtained were 10.53 h in winter, 10.20 h in autumn, 9.34 h in spring, and 8.76 h, in summer, ± 0.11 h (Table 2).

#### 3.3.5. Milk Composition (Milk Fat, Protein Fat, Fat/Protein Milk Ratio, Milk Urea)

The highest value for milk fat percentage (*p* < 0.05) occurred in autumn (3.49 ± 0.006), while the lowest value was observed in winter (3.38 ± 0.006), and intermediate and similar percentages were registered in spring and summer. Milk protein showed a biphasic behavior in relation to the season, with the highest values (*p* < 0.05) in autumn and winter (average = 3.163%) and the lowest ones (*p* < 0.05) in spring and summer (average = 3.111%). The highest value for the MF/MP ratio occurred in spring (1.10 ± 0.03 units), while the rest of the seasons showed the lowest values and were similar between them (from 1.07 to 1.08 units). Finally, milk urea also showed differences because of season, with the highest value (*p* > 0.05) in summer (11.4 ± 0.11 mg) and the lowest one (*p* < 0.05) in winter (10.1 ± 0.13 mg).

### 3.4. Milk Performance, HS, and Economic Impact at Animal, Farm, and Regional Levels

Based on the information generated in this study, if the average daily milk production by dairy cattle in the CL, in the comfort zone, was 35.9 L, and the said level of production was reduced to 31.8 L when dairy cows faced a high HS (i.e., >77 THI) when projecting such reduction (i.e., 4.1 L) to a lactation of 305 d per year, an annual reduction of 1250.5 L per cow will be expected.

## 4. Discussion

Our working hypothesis proposed that high THI levels will decrease milk production due to a depressed voluntary feed intake while a reduced feed conversion efficiency affects, in parallel, the comfort status of dairy cows in the CL. According to our research outcomes, milk yield, feed efficiency, and animal welfare were compromised as the environmental temperature increased (i.e., increased THIs). Therefore, our working hypothesis is not rejected. Such findings are of particular importance because the dairy cattle industry represents a significant economic activity in the CL, located in semi-desert northern Mexico. In fact, Mexico has one of the main clusters producing bovine milk in the American continent, specifically in the CL. Certainly, the dairy cattle production system not only generates a significant economic profit for dairy cow producers, but the system is also characterized because of its highly technical, up-to-date, and intensive production scheme, perfectly linked to a dairy industrialization structure, with national and international ramifications.

Being in a dry-hot semiarid region, however, marked differences are observed concerning the level of milk production because it is generated under an environment of thermal stress, affecting animal productivity, and with great discrepancies across seasons. The last is mainly generated by the increased proportion of days that those cows are outside the thermoneutral zone, characterized by more than 300 days with HS per year, and with a tendency to increase such environmental insult over time [26]. Therefore, as initially hypothesized, such HS decreased milk performance as well as fat and protein availability at the regional and national levels. In addition to such a productive scenario perfectly aligned with the significant economic importance generated by dairy cattle in this region, our results picture the massive challenge for dairy producers to guarantee the welfare and sustainability of the dairy cattle production system in the CL.

### 4.1. Milk Production either at the Farm Level or the Cow Level across THI and Seasons

Although the THI threshold for cows with high levels of production was relocated to 68 units [22], such a scenario did not occur in our study. Certainly, the highest milk production at either the farm level or the cow level was registered at 68–71 THI. The effects of HS on milk yield were observed from 72–77 THI onwards. According to Liu et al. [23] observing a 68 or 72 THI to declare an HS scenario is not required because of animals’ extremely diverse compensatory responses to face HS. Varied strategies to avoid or diminish the effects of HS by the farm managers could help to explain our study’s unaffected milk production level at 68–71 THI. Among the most plausible strategies are the adequacy of the facilities like providing sufficient and suitable shade [4,33,34,35,36,37,38] and evaporative cooling systems [4,34,39,40,41,42,43,44] which are, as stated by Roth [45], the most common strategies to lessen the effect of HS, especially in dry-hot arid environments. Other strategies consider nutritional modifications through the inclusion of dietary fiber, dietary fat, dietary microbial additives, vitamins, minerals, metal ion buffers, plant extracts, and other anti-stress additives. Both fiber and fats are easily available macronutrients that can lessen the negative impact of HS in dairy animals [23] as well as the reduction of very fibrous forages [46], the use of yeast in the diet [47,48], the inclusion of fats in the total mixed ration [49], the manipulation of the degradable protein [50], and the inclusion of K in cows’ diets [51]. It is important to point out that several of the above strategies are conducted on the farm under study to diminish the HS effects.

Another possible factor to explain our research outcomes involves the acclimation capacity of cattle born and raised in the region by the special synthesis of heat shock proteins (Hsps) when they were calves or heifers. The Hsps allow the animals to face HS when adults, promoting a better physiological response to environmental thermic challenges. According to Collier et al. [52], heat acclimation is a process that leads to a widening of the thermoregulatory range, enhancement of metabolic and signaling pathways, as well as protection against stressors other than heat by switching protective pathways to an alert state. Certainly, the process of acclimation involves changes in all levels of body organization, including the reprogramming of gene expression. Furthermore, although Hsps are primarily considered as being intracellular, Kristensen et al. [53] identified the presence of Hsp72 in plasma from Holstein–Friesian cows, concluding that, although Hsp72 is believed to be strictly stress-inducible, the finding of Hsp72 in plasma indicates that even apparently healthy individuals may experience extrinsic and (or) intrinsic stress.

In relation to the season of the year, the highest production was yielded in winter and the lowest in summer, both in TotMP and cowMP production. Ageeb and Hayes [54] reported that Holstein cows in Central Sudan had a loss of 2.7% in their production, while Imrich et al. [55] found a negative correlation between THI and milk yield (r = −0.65; *p* < 0.01). Besides, when dairy cows are exposed to HS for nearly 50% of all annual hours, a yearly depression in milk production of around 2072 kg/cow occurs [3]. As in our study, Becher et al. [56] reported diminished milk production due to HS associated with the summer months, although the negative consequences can continue during autumn. While Ageeb and Hayes [54] reported a noticeable milk reduction at the onset of the summer season, Du Preez et al. [57] commented that milk yield showed a decrease (i.e., 10% to 40%) in summer regarding winter. In our study, the annual losses per cow with respect to winter were 132.0 L in spring, 480.5 L in summer, and 259.4 L in autumn, for a total of 871.78 L.

### 4.2. Dry Matter Intake, Feed Conversion Efficiency, and Energy Corrected Milk across THI and Seasons

High THIs generate a DMI reduction; in our study, a reduction of DMI around 0.92 kg per cow occurred with 72–76 THI, while increases greater than 77 decreased up to 2.09 kg DMI as compared with THIs from <68 and 68–71. West et al. [58] reported a depressed DMI of 0.51 kg per unit of increase in THI beyond 72; Liu et al. [23] mention that DMI decreased by 4.0 kg/d when THI increased from 68 to 80. Regarding seasons, DMI varies across the seasons of the year; if considering the winter daily average DMI in our study (i.e., 24.2 kg) as 100% of DMI, spring, autumn, and summer registered a loss of 4%, 6%, and 13%, respectively (i.e., 0.9, 1.4, and, 3.1 kg). As environmental temperature increases in summer, the cow´s body temperature also increases, reducing their DMI to mitigate HS [59]. As in DMI, a similar trend occurred for the response variable FCE; as the THI augmented, the FCE was reduced (i.e., from 68–71 (1.62 units) to ≥77 (1.51 units). This is one of the most relevant variables to define milk production efficiency [59]. Under elevated environmental temperatures, DMI and milk yield decrease, but FCE is further depressed in heat-stressed cows than in cows kept in thermoneutral temperatures consuming the same amount of feed. Therefore, it is crucial to avoid large THI values since animals exposed to HS use a significant amount of energy to maintain body temperature and, consequently, less energy is diverted for milk production. The ECM also was affected for THI; the largest ECM occurred at the <68 and 68–71 levels, intermediate ECM values at 72–76 THI, while the lowest values were ≥77 THI. As expected, the best ECM occurred in winter, intermediate values in spring, with the lowest ECM values in summer and especially in autumn. An adequate intake of metabolizable energy is necessary to avoid milk depression in cows under HS [54]; any failure to maintain milk yield will occur with unbalances in the energy, protein, and lipid metabolism, depressing—in parallel—the immune response due to oxidative stress and inflammation [60].

### 4.3. Percentage of Milking Cows

The percentual reduction in lactating cows as the THI level increases will compromise the productive efficiency of the herd. As expected, the season also affected this variable, with the highest observed values in winter and spring. In well-managed herds, 85% of the cows are expected to be milked; any decrease in the proportion of milking cows denotes a lack of calving homogeneity across the year, which has been related to infertility problems due to increased HS [18]. A similar trend occurred regarding seasons; winter and spring had the highest value (i.e., 91.04%), while autumn and summer had the lowest values (i.e., 84.6 and 80.8%, respectively).

### 4.4. Dairy Cow Comfort

As the THI increases, a reduction in the resting time per cow was registered; the THIs <68 and 68–71 showed a similar (*p* > 0.05) lying time (i.e., 10.6 h). The LT decreased by 9.5 h and 8.5 h at THIs 72–76 and ≥77, respectively. Moreover, LT also differed among seasons (*p* < 0.05); the highest LT occurred in winter and autumn, regarding spring and summer. According to Tucker et al. [16], on average, lactating cows lie down daily for 8 to 13 h, within the range of our study. According to Heinicke et al. [61], LT decreased as the average daily THI increased; such LT decrease was even greater above the heat load threshold of 67 THI. According to Hut et al. [62], lactating cows spent, on average, 11.5 h/d lying; this value decreased by 6 min daily once the THI reached 56 and decreased gradually to 10.45 h/d when the THI ≥ 72. Also, Cook et al. [63] reported that LT decreased from the year’s coolest season to the hottest season. The LT is important in that it reflects animal welfare [64]; the less the LT, the more depressed the milk production, mainly due to a drop in DMI [65]. In general, the larger proportion of the rumination occurs while standing [16].

### 4.5. Milk Composition (Percentages of Fat and Protein in Milk and Quantity of Milk Urea)

Our results show that the milk fat percentage was low at <68 THI and higher at the other THI levels, and that, with respect to seasons, the highesst value was in autumn, and the lowest was in winter. These outputs do not agree with the results of several authors [9,19,66], who found a significant decrease in the levels of fat with increasing the THI, and [9] and [66] report a significantly lower fat content from spring to summer in the first case and in summer in the second one. However, other authors have reported inconsistent responses of fat content’s reaction to HS, with increasing, decreasing [67], or no change reports of fat percentage [22].

With respect to the protein milk percentage, we found an inverse relationship between protein milk and THI levels; in other words, the percentage diminishes conform the THI level increased. These results have correspondence with other reported early [9,19,66,68]. As ambient temperature increases and as body temperature concomitantly increases, cows decrease their feed intake to mitigate HS, thereby leading to a gradual decline in milk production and a change in milk content [23], then, failure to rescue milk yield and the changes in milk composition due to shifts in energy metabolism; protein catabolism; alterations in lipid metabolism due to endocrine alterations and immune response due to oxidative stress and inflammation are the major factors in this context [60]. However, Cowley et al. [68] attribute the reduction on milk protein concentration to specific downregulation or mammary protein, rather than a general reduction in milk synthesis.

Our results show that milk urea was higher at ≥77 THI and lower at 68–71 THI, with intermediate values at <68 and 72–76 THIs. Regarding season, the higher value was in summer and the lowest in winter, then, the quantities of milk urea are affected by THI and their seasonal distribution, as suggested by Muroya et al. [69]. Regarding the fact that HS and high humidity conditions have negative effects on the production of milk true proteins and increase the urea-N content in milk, the former agrees with Gernand et al. [70] who found that milk urea was quite constant within the THI range from THI 40 to 70, but when THI was higher than 70, milk urea substantially increased. Similarly, Cowley et al. [68] found increasing milk urea under HS conditions; they suspected that this increase is due to catabolized muscle tissue, a process that is intensified under HS conditions. Subsequently, in our results, the increase in milk urea as a consequence of a higher THI and season associated with THI could be the effect (not measured in our study) of plasma urea concentrations which are increasing, especially in cows at early lactation exposed to HS, as mentioned by Koch et al. [71].

At this point in the discussion, a reasonable question arises: How can we align our research outcomes to measure the impact of HS from the perspectives of the producer, the market, and the society? Unquestionably, this is a difficult undertaking. Nevertheless, projecting the said HS impact from an economic standpoint is a sensible way of sizing up the problem faced by the dairy cattle industry in the CL. Between 2020 and 2021, bovine milk production in the CL increased by 4% (i.e., 2613.58 to 2730.75 million (M) liters), generated by a daily increase in milk production from 7.16 to 7.48 M liters in those years. Interestingly, such an increase in the production level occurred despite the observed decrease in the CL milking cow census (i.e., 250,877 to 248,719). According to these results, while a 1% decrease in the number of milking cows occurred, a 4% increase in the volume of milk produced was observed [72]. The last occurred despite the significant thermal insult faced by dairy cattle in the CL, which exist for more than 300 days of the year [27].

When dimensioning, from an economic stance, how significant this thermal insult is at the regional level, some features related to the inventory and level of the regional dairy production must be considered. The CL concentrates 248,719 milking cows; based on the milk reduction observed in our study, an annual regional potential decrease of around 311 M liters of milk would be projected. Moreover, if the average payment to the producer is 0.4 USD per liter of milk, thermal stress potentially generates an annual economic loss for the producer of the CL equivalent to USD 233.2 M. Then, amplifying such projection at market value (i.e., 1 L = 1 USD), a critical but perilous stance, the potential annual economic loss in the industry and market for bovine milk in the CL, would be as high as USD 311 M.

Nevertheless, even more serious, albeit sensible, is to try to dimension such milk losses from the social and ethical points of view. While the right to food is recognized since 1948 in the Universal Declaration of Human Rights, thereafter, in the Millennium Declaration approved by the United Nations Assembly, a compromise was reached to halve the number of people suffering from hunger by the year 2015 [73]. Furthermore, the right to adequate food in the context of national food security was also agreed [74]. In this regard, Mexico does not produce enough milk to match national demand; more than 30% of the total milk consumed must be imported [75]. When projecting the economic and social impacts of the annualized loss of 311 M liters of milk due to HS in the CL, besides the detriment in the volume of milk available to society through the different market channels, we must also consider the significant loss of key nutrients, which similarly declines the national alimentary and nutritional security. Certainly, such loss in the milk volume generated by HS in the CL is more straightforwardly dimensioned when bearing in mind the associated losses, expressed in annual metric tons, of the following milk nutrients: fat (10,892.03), protein (10,251.32), lactose (14,415.92), minerals (2562.83), and vitamins (320.35). Interestingly, a total of 195,415.82 Gcal would not be available to society.

These numbers and trends undeniably highlight the massive challenge that—from environmental, biological, productive, economic, and social standpoints—the dairy cattle industry faces in the CL. Irrefutably, it is essential to intensify mitigation strategies to lessen the effects of HS on the regional dairy herd, such as the redesign of diets, the modification of infrastructure to increase the amount of shade and air circulation and, also, to improve the use of evaporative cooling, among others; however, other strategies in relationship with environment sustainability must be considered [76]. Still, these mitigation strategies have an essential requirement to hold an enhanced understanding of the main trends of the THI across years, seasons, months, and even throughout the day. Indisputably, a Big Data and Artificial Intelligence approach will increasingly become a constant to face the challenges from HS. Indeed, real-time capture systems (i.e., Big Data, minute-by-minute) of environmental information are essential to accomplish such a goal. Likewise, remote sensors (i.e., Artificial Intelligence) that quantify the body temperature of a lactating cow facing a thermal insult, together with other physiological and behavioral constants generated by the HS through wireless devices for continuous measurement, will certainly be essential to improve, modify, and expand HS mitigation strategies.

## 5. Conclusions

Despite the highly significant environmental challenge faced by dairy cows, the Comarca Lagunera in the arid north of Mexico produces more than 20% of the nation’s bovine milk volume. Said HS occurs for more than 300 days a year; such an environmental insult promotes the animal compensatory responses from both physiological and ethological standpoints. According to our research outcomes, HS provoked a lower feed-to-milk efficiency, reducing milk production both at the herd and cow levels, endangering not only animal welfare by reducing the resting hours but also decreasing the percentage of lactating cows, impacted by the effect of low fertility caused by HS. Even more critical, not only the economic losses at both the producer and the dairy industry levels but the accompanying nutritional losses at the society level undoubtedly impact the massive challenge that the dairy cattle industry in the Comarca Lagunera faces, from environmental, biological, productive, economic, and social standpoints. Finally, the proposal of measures to mitigate the thermal insult caused by HS on the bovine milk production and industrialization systems, in parallel with the quantification of how and when HS affects the chemical composition of milk in the region are, undeniably, pending assignments.

## Figures and Tables

**Table 1:** Descriptive statistics.

Variable	Mean	SD	Min	Max
Milk performance at farm level (L)	74,128.55	8652.31	54,752.00	90,787.00
Milk performance at cow level (L)	34.24	2.63	27.19	39.58
Dry Matter Intake (kg)	22.86	1.59	17.61	27.93
Feed-to-Milk Efficiency (units)	1.55	0.08	0.00	1.82
Energy-Corrected Milk (kg)	34.14	2.66	26.71	51.52
Milking Cows (%)	86.85	4.92	75.90	95.18
Lying Time (h)	9.72	0.99	6.30	11.87
Milk fat (%)	3.42	0.12	3.08	3.77
Milk protein (%)	3.14	0.07	2.97	3.33
Milk urea (mg)	10.78	1.79	6.41	17.37
THI (units)	72.76	5.42	35.46	83.23

**Table 2:** Least-square means ± standard error for daily volume of milk produced at farm (totMP) at cow level (cowMP); dry matter intake (DMI); Feed-to-Milk Efficiency (average Dry Matter Intake [DMI]; Feed Conversion Efficiency [FCE] and Energy-Corrected Milk [ECM]); Percentage of Milking Cows (MC%); Cow Comfortness (Lying Time; LT); milk fat; milk protein; and milk urea according to the THI level in a dairy farm from northern-arid Mexico (from 2016 to 2019).

Variables	Stress Level				
	<68 THI	68–71 THI	72–76 THI	≥77 THI	*p*-Value
Milk performance					
totMP (L)	76,114 ± 1038 ^ab^	78,886 ± 502 ^a^	75,095 ± 299 ^b^	66,584 ± 443 ^c^	<0.05
cowMP (L)	35.96 ± 0.30 ^a^	35.88 ± 0.15 ^a^	34.37 ± 0.09 ^b^	31.79 ± 0.13 ^c^	<0.05
Feed-to-milk efficiency					
DMI (kg)	23.58 ± 0.19 ^a^	23.77 ± 0.90 ^a^	22.76 ± 0.05 ^b^	21.59 ± 0.80 ^c^	<0.05
FCE (units)	1.575 ± 0.01 ^a^	1.562 ±.01 ^a^	1.558 ± 0.003 ^a^	1.514 ± 0.005 ^b^	<0.05
ECM (kg)	35.54 ± 0.30 ^a^	35.77± 0.15 ^a^	34.23 ± 0.09 ^b^	31.63 ± 0.13 ^c^	<0.05
Percentage of milking cows					
MC (%)	86.75 ± 0.64 ^b^	89.08 ± 0.31 ^a^	87.11 ± 0.18 ^b^	84.00 ± 0.27 ^c^	<0.05
Cow comfort					
LT (h)	10.71 ± 0.15 ^a^	10.53 ± 0.07 ^a^	9.46 ± 0.04 ^b^	8.52 ± 0.06 ^c^	<0.05
Milk composition					
Milk fat (%)	3.33 ± 0.02 ^a^	3.42 ±.01 ^b^	3.42 ± 0.004 ^b^	3.44 ± 0.01 ^b^	<0.05
Milk protein (%)	3.19 ± 0.01 ^a^	3.15 ± 0.003 ^b^	3.13 ± 0.002 ^c^	3.09 ± 0.003 ^d^	<0.05
Milk urea (mg)	10.9 ± 0.38 ^ab^	10.4 ± 0.15 ^b^	10.8 ± 0.09 ^ab^	11.0 ± 0.13 ^a^	<0.05

Different letters between columns show difference (*p* < 0.05). Data are presented as mean ± standard error of the mean.

**Table 3:** Least-square means ± standard error for volume of milk produced at farm (totMP), at cow level (cowMP), dry matter intake (DMI), Feed-to-Milk Efficiency (average Dry Matter Intake [DMI]; Feed Conversion Efficiency [FCE] and Energy-Corrected Milk [ECM]), Percentage of Milking Cows (MC%), Cow Comfortness, (Lying Time; LT), milk fat, milk protein, and milk urea, according to the season in a dairy farm from northern-arid Mexico (from 2016 to 2019).

Variables	Season				
	Spring	Summer	Autumn	Winter	*p*-Value
Milk performance					
totMP (L)	78,438 ± 307 ^b^	64,462 ± 305 ^d^	72,732 ± 305 ^c^	81,091 ± 309 ^a^	<0.05
cowMP (L)	35.20 ± 0.09 ^b^	31.37 ± 0.09 ^d^	33.80 ± 0.09 ^c^	36.65 ± 0.09 ^a^	<0.05
Food-to-milk efficiency					
DMI (kg)	23.29 ± 0.06 ^b^	21.18 ± 0.06 ^d^	22.79 ± 0.06 ^c^	24.23 ± 0.06 ^a^	<0.05
FCE (units)	1.56 ± 0.004 ^a^	1.53 ± 0.004 ^b^	1.53 ± 0.004 ^b^	1.56 ± 0.004 ^a^	<0.05
ECM (kg)	34.9 ± 0.10 ^b^	31.2 ± 0.10 ^d^	34.0 ± 0.10 ^c^	36.4 ± 0.10 ^a^	<0.05
Percentage of milking cows					
MC (%)	90.96 ± 0.12 ^a^	80.82 ± 0.12 ^c^	84.63 ± 0.12 ^b^	91.12 ± 0.12 ^a^	<0.05
Cow comfort					
LT (h)	9.34 ± 0.05 ^c^	8.76 ± 0.06 ^d^	10.20 ± 0.05 ^b^	10.53 ± 0.06 ^a^	<0.05
Milk composition					
Milk fat (%)	3.41 ± 0.006 ^b^	3.42 ± 0.006 ^b^	3.49 ± 0.006 ^a^	3.38 ± 0.006 ^c^	<0.05
Milk protein (%)	3.105 ± 0.003 ^b^	3.116 ± 0.003 ^b^	3.167 ± 0.003 ^a^	3.158 ± 0.003 ^a^	<0.05
Milk urea (mg)	10.9 ± 0.12 ^b^	11.4 ± 0.11 ^a^	10.6 ± 0.11 ^b^	10.1 ± 0.13 ^bc^	<0.05

Different letters between columns show difference (*p <* 0.05). Data are presented as mean ± standard error of the mean.

## Data Availability

The datasets used along with this research could be available from the corresponding author on reasonable request.

## References

[1] Nardone A., Ronchi B., Lacetera N., Ranieri M.S., Bernabucci U. (2010). Effects of Climate Changes on Animal Production and Sustainability of Livestock Systems. Livest. Sci..

[2] Bernabucci U., Biffani S., Buggiotti L., Vitali A., Lacetera N., Nardone A. (2014). The Effects of Heat Stress in Italian Holstein Dairy Cattle. J. Dairy Sci..

[3] St-Pierre N.R., Cobanov B., Schnitkey G. (2003). Economic Losses from Heat Stress by US Livestock Industries1. J. Dairy Sci..

[4] Becker C.A., Stone A.E. (2020). Graduate Student Literature Review: Heat Abatement Strategies Used to Reduce Negative Effects of Heat Stress in Dairy Cows. J. Dairy Sci..

[5] Armstrong D.V. (1994). Heat Stress Interaction with Shade and Cooling. J. Dairy Sci..

[6] Mader T.L., Frank K.L., Harrington J.A., Hahn G.L., Nienaber J.A. (2009). Potential Climate Change Effects on Warm-Season Livestock Production in the Great Plains. Clim. Chang..

[7] Kadzere C.T., Murphy M.R., Silanikove N., Maltz E. (2002). Heat Stress in Lactating Dairy Cows: A Review. Livest. Prod. Sci..

[8] Amundson J.L., Mader T.L., Rasby R.J., Hu Q.S. (2006). Environmental Effects on Pregnancy Rate in Beef Cattle1. J. Anim. Sci..

[9] Bouraouin R., Lahmar M., Majdoub A., Djemali M., Belyea R. (2009). Heat Stress in Tunisia: Effects on Dairy Cows and Potential Means of Alleviating It. S. Afr. J. Anim. Sci..

[10] Hernández A., Domínguez B., Cervantes P., Muñoz-Melgarejo S., Salazar-Lizán S., Tejeda-Martínez A. (2011). Temperature-Humidity Index (THI) 1917-2008 and Future Scenarios of Livestock Comfort in Veracruz, México. Atmosfera.

[11] Gantner V., Mijić P., Jovanovac S., Raguž N., Bobić T., Kuterovac K., Casasús I., Rogošiç J., Rosati A., Štokoviç I., Gabiña D. (2012). Influence of Temperature-Humidity Index (THI) on Daily Production of Dairy Cows in Mediterranean Region in Croatia. Animal Farming and Environmental Interactions in the Mediterranean Region.

[12] DeShazer J.A., Hahn G.L., Xin H. (2009). Chapter 1: Basic Principles of the Thermal Environment and Livestock Energetics. Agricultural and Biosystems.

[13] Mbuthia J.M., Mayer M., Reinsch N. (2022). A Review of Methods for Improving Resolution of Milk Production Data and Weather Information for Measuring Heat Stress in Dairy Cattle. Livest. Sci..

[14] Dikmen S., Cole J.B., Null D.J., Hansen P.J. (2013). Genome-Wide Association Mapping for Identification of Quantitative Trait Loci for Rectal Temperature during Heat Stress in Holstein Cattle. PLoS ONE.

[15] Tolkamp B.J., Haskell M.J., Langford F.M., Roberts D.J., Morgan C.A. (2010). Are Cows More Likely to Lie down the Longer They Stand?. Appl. Anim. Behav. Sci..

[16] Tucker C.B., Jensen M.B., de Passillé A.M., Hänninen L., Rushen J. (2021). Invited Review: Lying Time and the Welfare of Dairy Cows. J. Dairy Sci..

[17] Vasseur E., Rushen J., Haley D.B., de Passillé A.M. (2012). Sampling Cows to Assess Lying Time for On-Farm Animal Welfare Assessment. J. Dairy Sci..

[18] Hernández Cerón J. (2016). Fisiología Clínica de La Reproducción de Bovinos Lecheros.

[19] Ravagnolo O., Misztal I., Hoogenboom G. (2000). Genetic Component of Heat Stress in Dairy Cattle, Development of Heat Index Function. J. Dairy Sci..

[20] Hahn G.L., Mader T., Eigenberg R.A. (2003). Perspective on Development of Thermal Indices for Animal Studies and Management. EAAP Tech. Ser..

[21] Da Silva R.G., Morais D.A.E.F., Guilhermino M.M. (2007). Evaluation of Thermal Stress Indexes for Dairy Cows in Tropical Regions. Rev. Bras. De Zootec..

[22] Dikmen S., Hansen P.J. (2009). Is the Temperature-Humidity Index the Best Indicator of Heat Stress in Lactating Dairy Cows in a Subtropical Environment?. J. Dairy Sci..

[23] Liu J., Li L., Chen X., Lu Y., Wang D. (2019). Effects of Heat Stress on Body Temperature, Milk Production, and Reproduction in Dairy Cows: A Novel Idea for Monitoring and Evaluation of Heat Stress—A Review. Asian-Australas. J. Anim. Sci..

[24] Zimbelman R., Rhoads R., Rhoads M., Duff G., Baumgard L., Collier R. A Re-Evaluation of the Impact of Temperature Humidity Index (THI) and Black Globe Humidity Index (BGHI) on Milk Production in High Producing Dairy Cows. Proceedings of the 24th Annual Southwest Nutrition and Management Conference.

[25] Zhou M., Aarnink A.J.A., Huynh T.T.T., van Dixhoorn I.D.E., Groot Koerkamp P.W.G. (2022). Effects of Increasing Air Temperature on Physiological and Productive Responses of Dairy Cows at Different Relative Humidity and Air Velocity Levels. J. Dairy Sci..

[26] Pinto S., Hoffmann G., Ammon C., Amon T. (2020). Critical THI Thresholds Based on the Physiological Parameters of Lactating Dairy Cows. J. Therm. Biol..

[27] Rodriguez-Venegas R., Meza-Herrera C.A., Robles-Trillo P.A., Angel-Garcia O., Rivas-Madero J.S., Rodriguez-Martinez R. (2022). Heat Stress Characterization in a Dairy Cattle Intensive Production Cluster under Arid Land Conditions: An Annual, Seasonal, Daily, and Minute-To-Minute, Big Data Approach. Agriculture.

[28] Oltramari C.E., Pinheiro M.d.G., de Miranda M.S., Arcaro J.R.P., Castelani L., Toledo L.M., Ambrósio L.A., Leme P.R., Manella M.Q., Arcaro Júnior I. (2014). Selenium Sources in the Diet of Dairy Cows and Their Effects on Milk Production and Quality, on Udder Health and on Physiological Indicators of Heat Stress. Ital. J. Anim. Sci..

[29] Berry D.P., Crowley J.J. (2013). Cell Biology Symposium: Genetics of Feed Efficiency in Dairy and Beef Cattle. J. Anim. Sci..

[30] Tyrrell H.F., Reid J.T. (1965). Prediction of the Energy Value of Cow’s Milk. J. Dairy Sci..

[31] Lenth R.V., Buerkner P., Herve M., Love J., Miguez F., Riebl H., Singmann H. (2022). Package ‘Emmeans’. Estimated Marginal Means, Aka Least-Squares Means.

[32] Searle S.R., Speed F.M., Milliken G.A. (1980). Population Marginal Means in the Linear Model: An Alternative to Least Squares Means. Am. Stat..

[33] Herbut P., Angrecka S., Walczak J. (2018). Environmental Parameters to Assessing of Heat Stress in Dairy Cattle—A Review. Int. J. Biometeorol..

[34] Gebremedhin K.G. (2012). Heat Stress and Evaporative Cooling. Environmental Physiology of Livestock.

[35] Cincović M., Majkić M., Belić B., Plavša N., Lakić I., Radinović M. (2017). Thermal Comfort of Cows and Temperature Humidity Index in Period of 2005–2016 in Vojvodina Region (Serbia). Acta Agric. Serbica.

[36] Theusme C., Avendaño-Reyes L., Macías-Cruz U., Correa-Calderón A., García-Cueto R.O., Mellado M., Vargas-Villamil L., Vicente-Pérez A. (2021). Climate Change Vulnerability of Confined Livestock Systems Predicted Using Bioclimatic Indexes in an Arid Region of México. Sci. Total Environ..

[37] Gantner V., Mijić P., Kuterovac K., Solić D., Gantner R. (2011). Temperature-Humidity Index Values and Their Significance on the Daily Production of Dairy Cattle. Mljekarstvo.

[38] Collier R.J., Renquist B.J., Xiao Y. (2017). A 100-Year Review: Stress Physiology Including Heat Stress. J. Dairy Sci..

[39] Spiers D.E., Spain J.N., Ellersieck M.R., Lucy M.C. (2018). Strategic Application of Convective Cooling to Maximize the Thermal Gradient and Reduce Heat Stress Response in Dairy Cows. J. Dairy Sci..

[40] Tresoldi G., Schütz K.E., Tucker C.B. (2019). Cooling Cows with Sprinklers: Effects of Soaker Flow Rate and Timing on Behavioral and Physiological Responses to Heat Load and Production. J. Dairy Sci..

[41] Tresoldi G., Schütz K.E., Tucker C.B. (2018). Cooling Cows with Sprinklers: Timing Strategy Affects Physiological Responses to Heat Load. J. Dairy Sci..

[42] Shiao T.F., Chen J.C., Yang D.W., Lee S.N., Lee C.F., Cheng W.T.K. (2011). Feasibility Assessment of a Tunnel-Ventilated, Water-Padded Barn on Alleviation of Heat Stress for Lactating Holstein Cows in a Humid Area1. J. Dairy Sci..

[43] Fournel S., Ouellet V., Charbonneau É. (2017). Practices for Alleviating Heat Stress of Dairy Cows in Humid Continental Climates: A Literature Review. Animals.

[44] Turner L.W., Chastain J.P., Hemken R.W., Gates R.S., Crist W.L. (1992). Reducing Heat Stress in Dairy Cows Through Sprinkler and Fan Cooling. Appl. Eng. Agric..

[45] Roth Z. (2020). Reproductive Physiology and Endocrinology Responses of Cows Exposed to Environmental Heat Stress—Experiences from the Past and Lessons for the Present. Theriogenology.

[46] Kanjanapruthipong J., Homwong N., Buatong N. (2010). Effects of Prepartum Roughage Neutral Detergent Fiber Levels on Periparturient Dry Matter Intake, Metabolism, and Lactation in Heat-Stressed Dairy Cows. J. Dairy Sci..

[47] Bruno R.G.S., Rutigliano H.M., Cerri R.L., Robinson P.H., Santos J.E.P. (2009). Effect of Feeding Saccharomyces Cerevisiae on Performance of Dairy Cows during Summer Heat Stress. Anim. Feed. Sci. Technol..

[48] Perdomo M.C., Marsola R.S., Favoreto M.G., Adesogan A., Staples C.R., Santos J.E.P. (2020). Effects of Feeding Live Yeast at 2 Dosages on Performance and Feeding Behavior of Dairy Cows under Heat Stress. J. Dairy Sci..

[49] Chan S.C., Huber J.T., Chen K.H., Simas J.M., Wu Z. (1997). Effects of Ruminally Inert Fat and Evaporative Cooling on Dairy Cows in Hot Environmental Temperatures. J. Dairy Sci..

[50] Kaufman J.D., Pohler K.G., Mulliniks J.T., Ríus A.G. (2018). Lowering Rumen-Degradable and Rumen-Undegradable Protein Improved Amino Acid Metabolism and Energy Utilization in Lactating Dairy Cows Exposed to Heat Stress. J. Dairy Sci..

[51] Mallonée P.G., Beede D.K., Collier R.J., Wilcox C.J. (1985). Production and Physiological Responses of Dairy Cows to Varying Dietary Potassium During Heat Stress. J. Dairy Sci..

[52] Collier R.J., Collier J.L., Rhoads R.P., Baumgard L.H. (2008). Invited Review: Genes Involved in the Bovine Heat Stress Response. J. Dairy Sci..

[53] Kristensen T.N., Løvendahl P., Berg P., Loeschcke V. (2004). Hsp72 Is Present in Plasma from Holstein-Friesian Dairy Cattle, and the Concentration Level Is Repeatable across Days and Age Classes. Cell Stress Chaperones.

[54] Ageeb A.G., Hayes J.F. (2000). Genetic and Environmental Effects on the Productivity of Holstein-Friesian Cattle under the Climatic Conditions of Central Sudan. Trop. Anim. Health Prod..

[55] Imrich I., Toman R., Pšenková M., Mlyneková E., Kanka T., Mlynek J., Pontešová B. (2021). Effect of Temperature and Relative Humidity on the Milk Production of Dairy Cows. Sci. Technol. Innov..

[56] Becker C.A., Collier R.J., Stone A.E. (2020). Invited Review: Physiological and Behavioral Effects of Heat Stress in Dairy Cows. J. Dairy Sci..

[57] Du Preez J.H., Hattingh P., Giesecke W., Eisenberg B.E. (1990). Monthly Temperature-Humidity Index Mean Values and Their Significance in the Performance of Dairy Cattle. Onderstepoort J. Vet Res..

[58] West J.W., Mullinix B.G., Bernard J.K. (2003). Effects of Hot, Humid Weather on Milk Temperature, Dry Matter Intake, and Milk Yield of Lactating Dairy Cows. J. Dairy Sci..

[59] Bach A., Terré M., Vidal M. (2020). Symposium Review: Decomposing Efficiency of Milk Production and Maximizing Profit. J. Dairy Sci..

[60] Nogoy K.M.C., Park J., Chon S.I., Sivamani S., Park M.J., Cho J.P., Hong H.K., Lee D.H., Choi S.H. (2021). Precision Detection of Real-Time Conditions of Dairy Cows Using an Advanced Artificial Intelligence Hub. Appl. Sci..

[61] Heinicke J., Hoffmann G., Ammon C., Amon B., Amon T. (2018). Effects of the Daily Heat Load Duration Exceeding Determined Heat Load Thresholds on Activity Traits of Lactating Dairy Cows. J. Therm. Biol..

[62] Hut P.R., Scheurwater J., Nielen M., van den Broek J., Hostens M.M. (2021). Sensor-Based Behavioral Patterns of Dairy Cows. J. Dairy Sci..

[63] Cook N.B., Mentink R.L., Bennett T.B., Burgi K. (2007). The Effect of Heat Stress and Lameness on Time Budgets of Lactating Dairy Cows. J. Dairy Sci..

[64] EFSA (2009). Effects of Farming Systems on Dairy Cow Welfare and Disease.

[65] Munksgaard L., Jensen M.B., Pedersen L.J., Hansen S.W., Matthews L. (2005). Quantifying Behavioural Priorities—Effects of Time Constraints on Behaviour of Dairy Cows, Bos Taurus. Appl. Anim. Behav. Sci..

[66] M’Hamdi N., Darej C., Attia K., El Akram Znaidi I., Khattab R., Djelailia H., Bouraoui R., Taboubi R., Marzouki L., Ayadi M. (2021). Modelling THI Effects on Milk Production and Lactation Curve Parameters of Holstein Dairy Cows. J. Therm. Biol..

[67] Nasr M.A.F., El-Tarabany M.S. (2017). Impact of Three THI Levels on Somatic Cell Count, Milk Yield and Composition of Multiparous Holstein Cows in a Subtropical Region. J. Therm. Biol..

[68] Cowley F.C., Barber D.G., Houlihan A.V., Poppi D.P. (2015). Immediate and Residual Effects of Heat Stress and Restricted Intake on Milk Protein and Casein Composition and Energy Metabolism. J. Dairy Sci..

[69] Muroya S., Terada F., Shioya S. (1997). Influence of Heat Stress on Distribution of Nitrogen in Milk. Nihon Chikusan Gakkaiho.

[70] Gernand E., König S., Kipp C. (2019). Influence of On-Farm Measurements for Heat Stress Indicators on Dairy Cow Productivity, Female Fertility, and Health. J. Dairy Sci..

[71] Koch F., Lamp O., Eslamizad M., Weitzel J., Kuhla B. (2016). Metabolic Response to Heat Stress in Late-Pregnant and Early Lactation Dairy Cows: Implications to Liver-Muscle Crosstalk. PLoS ONE.

[72] (2022). Secretaría de Agricultura y Desarrollo Rural Livestock Production in the Region Lagunera.

[73] UN (1948). Universal Declaration of Human Rights.

[74] FAO (2004). The State of Food and Agriculture.

[75] SIAP Anuario Estadístico de La Producción Ganadera. https://www.gob.mx/cms/uploads/attachment/file/744950/Inventario_2021_bovino_para_leche.pdf.

[76] Navarrete-Molina C., Meza-Herrera C.A., Ramirez-Flores J.J., Herrera-Machuca M.A., Lopez-Villalobos N., Lopez-Santiago M.A., Veliz-Deras F.G. (2019). Economic Evaluation of the Environmental Impact of a Dairy Cattle Intensive Production Cluster under Arid Lands Conditions. Animal.

